# Evaluating the Impact of Integrative Art Therapies on Psychological Well-Being in Pediatric Oncology: A Single-Group Pre–Post Study

**DOI:** 10.3390/nursrep16040125

**Published:** 2026-04-09

**Authors:** Farzana Ashraf, Urooj Sadiq, Shahnila Tariq, Bushra Awan, Selma Yıldırım, Carlos Laranjeira, Murat Yıldırım

**Affiliations:** 1 Department of Humanities, COMSATS University, Lahore 54000, Pakistan; farzana.ashraf@cuilahore.edu.pk; 2 Department of Professional Psychology, Bahria University, Lahore Campus, Lahore 75260, Pakistan; urooj.bulc@bahria.edu.pk; 3 Department of Psychology, University of Management and Technology, Lahore 54770, Pakistan; shahnila.tariq@umt.edu.pk; 4 Shaukat Khanum Memorial Cancer Hospital & Research Centre, Lahore 54000, Pakistan; awanbushra@hotmail.com; 5 Graduate School of Natural and Applied Sciences, Kafkas University, Kars 36100, Türkiye; sselmayyildirim@gmail.com; 6 School of Health Sciences, Polytechnic University of Leiria, Campus 2, Morro do Lena, Alto do Vieiro, Apartado 4137, 2411-901 Leiria, Portugal; 7 Centre for Innovative Care and Health Technology (ciTechCare), Polytechnic University of Leiria, Campus 5, Rua das Olhalvas, 2414-016 Leiria, Portugal; 8 Comprehensive Health Research Centre (CHRC), University of Évora, 7000-801 Évora, Portugal; 9 Department of Psychology, Faculty of Science and Letters, Agri Ibrahim Cecen University, Agri 04100, Türkiye; 10 Psychology Research Center, Khazar University, Baku 1009, Azerbaijan

**Keywords:** pediatric cancer patients, positive self-image, negative self-image, mental health problems, psychological well-being

## Abstract

**Background:** Art therapy is an experiential, non-threatening intervention, used especially with children. The current study aimed to explore the effect of integrative art therapy on the psychological well-being (mental health and self-perception) of pediatric cancer patients. **Methods:** Using a single-group pre–post research design, each therapy session was individually administered to participants for approximately 45 min. Fourteen participants (Boys = 9, Girls = 5) were recruited from the inpatient oncology unit at Shaukat Khanum Memorial Cancer Hospital & Research Centre over two months. The age range was from 5 years to 13 years (*M* = 7.95; *SD* = 1.65). Mental health, including physical and emotional symptoms associated with cancer, was assessed using the Edmonton Symptom Assessment Scale-Revised, while self-perception was measured with the House Tree Person projective drawing test. **Results:** The integrative art therapy model significantly improved positive self-image (*F* = 16.77, *p* < 0.01) and reduced negative self-image (*F* = 99.11, *p* < 0.01) and mental health problems from the baseline to the second and third phases (*F* = 19.50, *p* < 0.01). **Conclusions:** This integrative approach demonstrates its potential as an effective method to enhance self-perception, alleviate mental health challenges, and improve overall quality of life.

## 1. Introduction

Cancer is a chronic disease [1], the second deadliest disease globally, surpassed only by cardio-vascular diseases [2]. Cancer incidence has risen by 33% within the past decade [3]. The prevalence and manifestation of cancer vary substantially between pediatric and adult populations. Among all types of cancer, leukemia is the deadliest type, with the highest mortality rate in children [2]. Most patients experience moderate to severe physical pain during cancer [4], and the treatment of this disease, such as chemotherapy, is painful. In addition to the physical pain (medical processes, hospitalization, physical complications, and missed classes), they may also suffer from unimaginable mental health problems, not only due to the disease but also the treatment process [5].

The psychological effects of cancer can lead to psychiatric issues in patients [6]. These mental health challenges may include stress, depression, anxiety about death, fear of recurrence, separation anxiety, hopelessness, guilt, low self-esteem, negative self-image, dissatisfaction, embarrassment, and various other emotional turmoil [1,7].

These physical and mental health problems lead to an increase in the severity of symptoms affecting the physical, social, and emotional functioning of children [8] and the mortality rate as well [9]. Most patients do not follow psychological treatment in combination with medical treatment (due to socioeconomic status, lack of motivation, or lack of insight), which leads to poor quality of life. In addition to pharmacological treatment, psychological treatment is also beneficial for such patients [10,11]. Common therapies applied for the treatment of cancer include cognitive behavioral therapy, mindfulness-based cognitive therapy, acceptance and commitment therapy, and rational emotive behavior therapy. Art therapy is also emerging as an effective treatment [12,13].

Art therapy is an experiential, non-threatening intervention, used especially to treat children [14]. It is a form of psychotherapy using a creative process through art materials and resulting imagery to help individuals foster personal growth and reduce anxiety by expressing emotions. The therapist engages the child with painting, sculpting, visual imagery, and clay, helping them articulate their emotions, memories, and feelings, thereby fostering introspection and the development of new coping strategies through this creative and expressive progression [15]. This intervention reveals insights for the treatment of children’s psychological problems, such as low self-esteem, self-awareness problems, emotional dysregulation or resilience, and poor social skills [14,16].

Art therapy is a form of expressive art therapy, which represents a multidimensional, psychologically grounded approach that integrates diverse artistic modalities. These therapies are broader in scope, essentially sensory-based and action-oriented therapeutic alliances that combine diverse modes of visual arts (photography, sculpting/clay making, collage work, drawing, and painting), performing arts (role playing, dance/movement, music, and improvisation), and literary arts (storytelling, poetry writing, and journaling) within a single therapeutic practice to promote self-awareness, emotional regulation, and self-perception [17]. The continuum of art therapy involves a vast range of expressive arts such as ceramics, knitting, sketching, painting, clay playing, videography, stone carving, etc. [17], which are used effectively to treat psychological and physiological, social and behavioral problems such as cognitive impairment, anxiety, immune function, depression, and language impairment [18,19].

Keeping in view the feasibility of assessment and participants’ characteristics (age, cultural familiarity with art, academic background, diagnosis, stage of treatment, and emotional and cognitive functioning), the visual modality of art therapy, including playdough and collage making, and drawing, was selected. The rationale for using art therapy is that it helps patients actively create artwork, thereby providing opportunities for physical health management [20]. According to studies, art therapy is beneficial for health prevention, health promotion, and management of patients at different stages of life [21], especially in cancer patients [22]. Art therapy is beneficial for cancer patients in reducing hopelessness and emotional despair during the treatment and post-treatment stages.

Play therapy is when the therapist comes to the child’s level and talks to them in their language via play or toys. It encourages the child to express, understand, and accept their emotions and feelings [23]. Children who suffer from abuse, violence, and disrupted attachment can be healed through this approach [24]. It was strongly suggested [25] that play therapy is beneficial in improving social acceptance, hope, and self-esteem in children facing cancer. A combination of play and art therapy was used for mothers and children facing domestic violence [26]. The results showed that this combination was effective, but the study was not experimental. Additionally, play therapy has not previously been combined with art therapy for cancer patients, so exploring this combination’s effects in an experimental study would be beneficial.

Collage art and painting are effective approaches frequently used in Art therapy; in collage, individuals create artwork by selecting and arranging images and words that hold personal significance, whereas in painting, they express themselves through drawing or painting using various colors and materials. Both techniques serve as effective therapeutic interventions, focusing on a person’s self-care, psychology, and emotional well-being [27,28]. They help to improve emotional expression, communication, concentration, introspection, self-esteem, and social integration, thus reducing anxiety, obsessive symptoms, depression, and other psychopathologies [29,30,31]. They also serve the subjects by helping them explore fragmented images and perspectives of their experiences, potentials, and limitations. According to studies, they can be used as a complementary treatment for cancer patients [32,33,34], but the specific effect of collage therapy on children facing cancer warrants studies focused on this approach.

Playdough art is a modality of art therapy that facilitates the processing of internal experiences, thoughts, and emotions. Individuals construct thought bubbles using playdough, subsequently reconfigured with the guidance of a therapist. This process enables individuals to articulate and confront experiential narratives, distressing emotions, and cognitions, thereby fostering regulation of thoughts, experiences, and emotions [35]. It promotes healing through improved self-awareness, self-esteem, self-confidence, problem-solving, and personal growth in patients facing medical and psychological problems [36]. The effectiveness of the playdough approach in play therapy has not been assessed in cancer patients, especially children, so it would be beneficial to explore the generalization of the healing effect of all types of play therapy in cancer patients.

Art therapy is effectively used to treat physiological, social, and behavioral problems in addition to mental disorders such as cognitive impairment, immune function, and language impairment [19]. It also helps in the rehabilitation of post-stroke patients [37], dementia [38], and Parkinson’s disease [39]. The rationale for using art therapy is that it helps patients actively create artwork, thereby promoting physical health management by reducing hopelessness and anxiety [20,40].

Art therapy has a crucial role in improving the mental well-being of children experiencing chronic illnesses such as cancer [41]; it fosters catharsis via expression of emotional needs through artwork [35] and also improves quality of life by empowering children and teaching them new coping strategies to face the challenges related to cancer and its treatment [42]. Integrating art therapy into their treatment plan creates a more comprehensive and compassionate approach that addresses their emotional and psychological needs.

### 1.1. Research Gaps

In past studies [12,34,41], a diverse range of art therapies has been shown to reduce physical and psychological burden (anxiety, depression, low self-esteem, poor social skills, emotional dysregulation, impaired immune function) and improve treatment adherence in pediatric cancer patients. Yet none of these studies examined the efficacy of integrative art therapy modalities specifically in pediatric oncology patients. Despite these findings from past studies, an integrative model combining art therapy and play therapy has not yet been explored. Bridging this gap can revolutionize the care provided to pediatric patients. The paucity of literature on art therapy is greater for children than for adults due to the feasibility of assessment modes. Children are more expressive and receptive to creative modalities than adult cancer patients, as art therapies provide a non-verbal channel for expressing complex feelings and emotions. Children’s inherently imaginative and playful nature aligns with natural modes of exploration, allowing them to tap into their inner resources and resilience.

The rationale for conducting this research is to provide advocacy substance to the facts and statistics that support the effectiveness of art therapy. This research produces powerful information that has not been explored directly and indigenously before. Art psychology investigates human roles and examines the relationship between the unconscious, conscience, and feelings [43]. Psychologists have proven that art therapy is an effective means of exploring the unconscious mind. Children often struggle to verbalize their emotions, anxieties, and fears. Prolonged intense treatment puts cancer patients at the highest risk for psychological issues. Researchers have shown the significant impact of such treatment modalities in gaining access to a patient’s pent-up emotions. In Pakistan, such art therapy could be more varied. Therefore, given the effectiveness of art therapy, more and more research needs to be conducted indigenously to provide a framework for clinical practice.

### 1.2. Hypotheses of the Study

1. Art therapy alleviates mental health issues in children with cancer.
2. Integrative art therapies enhance positive self-image in pediatric cancer patients.
3. Art therapies reduce negative self-perception in children with cancer.

## 2. Materials and Methods

### 2.1. Study Design and Sample

The intervention followed a single-group pre–post research design. The intervention consisted of pretest and posttest measures administered individually to the participants. Initially, the aim was to randomly recruit thirty participants from the inpatient oncology unit at Shaukat Khanum Memorial Cancer Hospital & Research Centre (SKMCH) over two 2-month periods, and distribute them equally into control and experimental groups. This sample size was determined using G*Power (version 3.1.9.7) analysis with an estimated alpha level of *p* < 0.05 and an effect size of 0.80 (Medium to large). However, this sample size proved difficult to obtain given the unavailability of the required number of participants at SKMCH. Most patients were from Non-Urdu-speaking regions of Pakistan; therefore, the targeted population pool was markedly reduced. Therefore, it was decided to exclude the control group due to the expected low and disproportionate number of participants in the control and experimental groups, thus leaving only the experimental group. An initial sample of eighteen participants was reduced as two expired during the baseline phase; one underwent arm amputation during the second phase of the research; and one participant left the city during the first phase of the research. In the end, fourteen participants (Boys = 9, Girls = 5) completed all three study phases, which also met the minimum sample size as determined by the G*Power analysis with an estimated alpha level of *p* < 0.05 and an effect size of 0.80 (Medium to large) for a single-group pre–post study. The age range was from 5 years to 13 years (*M* = 7.95, *SD* = 1.65). Patients with intact cognitive function were selected (according to the reports of play therapists who regularly attended their sessions).

**Inclusion and exclusion criteria:** The following criteria were used to assess the empirical impact of the intervention. For this purpose, an online hospital information system was used to obtain complete details of the participants. Only stage I cancer patients who could understand the Urdu language and could be contacted for weeks for intervention-based research were selected. Participants were also matched on socio-economic status, as SKMCH is a charity hospital and has very stringent criteria for validating and treating patients with low socio-economic status. Only children at stage I of cancer and with an active treatment phase were selected. Participants with other co-morbid diseases and those with any physical disability/impairment were excluded from the sample. Furthermore, children with varied disease progression were excluded. Children who could speak and understand Urdu and were not diagnosed or identified with any other disability or emotional or behavioral problems before the diagnosis of cancer were included. Those who did not fulfill the inclusion criteria were excluded.

The study followed the Transparent Reporting of Evaluations with Nonrandomized Designs (TREND) guidelines [44].

### 2.2. Assessment Measures

**Demographic information sheet:** The self-constructed demographic information sheet was used to collect personal information, including family history, onset of illness, age, education, joint/nuclear family system, siblings, birth order, and the number of family visits. These variables were also used as covariates in the data analysis.

**Edmonton Symptom Assessment:** The instrument used to measure mental health (physical and emotional symptoms) associated with cancer was the Edmonton Symptom Assessment Scale-Revised (ESAS-r). The original tool was developed by Alberta Health Services in Edmonton [45]. The ESAS was designed to allow the patient or their family caregiver to self-administer the tool. Therefore, the patient should be taught how to complete the scale. The patient’s assessment of severity is the gold standard for symptom assessment. The modified ESAS-r is a 9-item patient-reported numeric symptom scale developed for use in palliative care. It has been validated in other populations, including cancer inpatients. In the ESAS-r, patients rate the severity of each of the following nine symptoms on a 0–10 scale measuring pain, tiredness, nausea, depression, anxiety, drowsiness, lack of appetite, well-being, and shortness of breath, with 0 meaning that the symptom is absent and 10 that it is of the worst possible severity. Ideally, patients fill out their own ESAS. However, as patients aged 5 to 13 years were selected for the present study, it was difficult for them to rate their current condition. ESAS was completed twice, once by the patient and once by the attendant.

The sum of the patient’s responses to these nine symptoms is the global ESAS-r distress scores [46]. The Alberta Health Services [45] has already translated the scale into Urdu. It has been translated into various languages and is freely available on the web, with appropriate acknowledgement of its source. It has been validated in various languages. Reliability, good internal consistency (Cronbach’s alpha 0.86), and the equivalence of the Urdu, Spanish, and Korean versions were between 0.71 and 0.94 [47,48]. ESAS-r was administered at baseline and after each art therapy session.

**The integrative model of art therapy:** Different art materials allow different types of emotional statements [49], leading to psychological well-being. Therefore, integrated model Art Therapy (AT) techniques were applied. Based on prior literature, the current study used three major art modalities, i.e., Collage Making, Free Drawing, and Clay Art. Collage Making is the process of creating collages using various materials (e.g., pictures, shapes, scissors, glue, etc.). In Clay Art, participants use clay to create three-dimensional representations of experiences, feelings, and thoughts, promoting self-reflection and tactile expression. Free Drawing is characterized by unstructured drawings using various media (e.g., paint, markers, pencils), which facilitate spontaneous emotional release and expression. Participants were administered these interventions once, with a one-week gap between the three sessions. Each session lasted from 25 to 45 min in the playroom. To ensure internal validity, the same order of art modalities was administered to each participant. In addition, art therapies were administered by the specialized and experienced Play Therapist who met minimum qualifications, certifications, and requirements to administer these therapies. Semi-structured protocols outlining session goals, activities, and emerging themes were used. Below is the schedule and rationale of the art therapies used as an intervention in the group.

***Session 1* → *Collage Making*:** Participants were given magazine pictures, decorative shapes, yarns, feathers, beads, and jewels for collage-making. These materials are perceived as non-threatening for patients who are reluctant to engage in artistic expression. This technique is therapeutic in a way, as cancer patients experience it as a process of taking the fragmented pieces of their lives and bringing them together into some sense of order [46].

***Session 2* → *Free Drawing*:** In the second phase, patients were given drawing materials such as pencils, colored pencils, charcoal pencils, oil crayons, marker pens, and pastels. This method is remedial in that it gives patients a variety of controlled media to outline their thoughts on paper. Paints, by contrast, being more fluid, can create strong expressions that express more feelings [46].

***Session 3* → *Clay*:** Clay is another excellent choice because it is a tactile, sensual material that brings a third dimension to the expression. Therefore, participants were allowed to play freely with playdough for approximately half an hour to express their emotions fully [46]. During each creative process session, the therapist facilitated insight, offered suggestions, validated the participants’ feelings, and listened actively.

***Assessment of perception of self*.** Since good mental health impacts self-image, the participant was also asked to draw themself on a blank page, once during the baseline phase and then after each session, to assess the efficacy of the art therapy. To assess a person’s self-image, the “Person” part of the House Tree Person (HTP) was used and interpreted in terms of positive and negative symptoms using an HTP manual. The projective mode of assessment was selected, given the absence of culturally standardized objective personality measurement tools. Projective measures tap more effectively into the unconscious feelings and thoughts that participants, especially children, find difficult to express. Additionally, rich, deep-rooted data on participants’ experiences facilitates understanding complex emotions that are hard to quantify in objective, self-reported measures. The instructions for administering HTP and the participant’s present condition were also considered when interpreting the results of each session.

### 2.3. Procedure

Demographic information was collected after a patient expressed interest in participating in the AT study. Informed consent was obtained, and the baseline assessment was completed. This study was carried out in five phases, as described below. A play therapist who has worked at Shaukat Khanum Memorial Cancer Hospital & Research Center for five years and has sound knowledge of AT developed therapy plans with each participant. Each subject’s nurse was consulted before the session to minimize potential interruptions. The subject’s attendant was instructed that the session would last approximately 45 min. Informed consent was obtained from the attendants, who were briefed about the purpose of the study and the side effects of chemotherapy. The language that was most easily understandable for the attendant was used. They were also told that the intervention would have no harmful effects and that they could withdraw from the research at any time. A few attendants wanted to know about the outcome of the intervention, and be sure they would receive results after completion of the study. The therapist also assisted subjects in selecting their subject matter and media. Children were given different media for expression and freely selected any medium to express themselves fully and freely, thereby helping them regain their mental health. The intervention plan took almost two months for its applicant.

**Phase 1: Baseline phase.** Hospitalized patients (*N* = 14) were invited to participate in the study. Participation was voluntary, and participants were not pressured. Each participant was provided with an information sheet outlining the purpose and objectives. The Edmonton Symptom Assessment Scale (ESAS-r) was administered as a baseline assessment. This scale was translated (largely pictorial, with non-technical language) for assessment. In addition to the above scale, the patient was asked to draw “themselves” on a blank page to assess their self-perception.

**Phase 2: Therapy plan.** The selected participants were treated with an integrated model of art therapy. The therapy plan sessions lasted one month and one week.

**Phase 3: Posttest.** A test using the Edmonton Symptom Assessment Scale (ESAS-r) was administered at the end of all therapeutic sessions.

### 2.4. Data Analysis

Data was analyzed using SPSS version 22. Means, standard deviations, frequencies, and percentages were estimated for the descriptive characteristics of participants. Inferential statistics included repeated-measure ANCOVA.

### 2.5. Ethical Considerations

The Research Ethics Committee at COMSATS University Islamabad Lahore Campus (reference number: HUM/CUI/LHR:109) granted ethical approval to conduct the study. Parents/guardians of the participants were well-informed about the purpose and objectives of the study, as well as the confidentiality and privacy of the information provided. They were also informed that participant information would only be used for academic/research purposes. All procedures performed in this study involving human participants were in accordance with institutional and national research ethics standards. As the participants were children aged 5 and 13 years (*M* = 7.95, *SD* = 1.65), informed consent was obtained from their legal guardians before participation. Children were also provided with age-appropriate information about the study and asked to assent to their involvement. After this, an information sheet was provided that outlined the study’s objective and its benefits. Participants were informed that participation in the study was voluntary. Attendants were assured that the treatment would not be adversely influenced if their children decided to participate. All participants were treated with respect and fairness and assured that they would not be harmed as a result of their participation in the study. To ensure confidentiality and privacy, participants were assured that their information would remain confidential and that their identities would not be revealed in any publications or presentations in the research dissemination process.

## 3. Results

Of the 30 regular patients approached, only 14 completed the intervention program (Boys = 9, Girls = 5). The study participants diagnosed with stage I cancer who could be contacted during the week-long intervention-based research were selected. Therefore, it was decided to exclude the control group and retain only the experimental group to which the intervention could be applied. Initially, N = 18 were approached, but two participants expired during the baseline phase, one left the city during the first phase of the research, and one underwent arm amputation during the second phase of the research. Thus, the final sample comprised 14 participants with various forms of cancer, all diagnosed with stage I of cancer within the last 6 months, and staying in Lahore city, Pakistan. They lived with at least one family member and completed all three study phases. The age range of participants was from 5 years to 13 years (*M* = 7.95, *SD* = 1.65). Patients with intact cognitive power were selected. Mean, standard deviations, frequencies, and percentages were obtained. Moreover, the average duration of diagnosis was 4.43 (*SD* = 1.50) months, and the average duration of treatment was 2.01 (*SD* = 13.3) months. Fifty-seven percent of participants (N = 8) have 4 or fewer siblings, and 43% (N = 6) have 5 to 8 siblings. One participant was firstborn, 1 was lastborn, and 12 were middleborn; none of the participants was an only child. Out of 14, 64% (n = 9) were living in a separate family system, while 36% (n = 5) were living in a joint family system.

**Figure 1:**
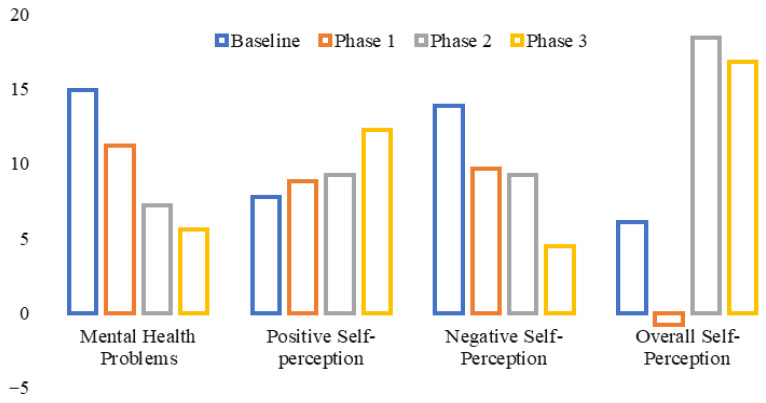
Comparison of mental health problems and self-perception in all phases.

Repeated-measures ANCOVA was applied to examine the efficacy of art therapy across sessions while controlling the confounding influence of participants’ age, gender, family system, number of siblings, birth order, duration of diagnosis, and duration of treatment. None of the demographic variables affected or interacted significantly with the art therapy and mental health issues measures. The two-month intervention programs using an integrative model of art therapy had a statistically significant effect on the positive self-perception of children with cancer (*F* (1, 13) = 16.7, *p* = 0.001), as evident from Table 1 and Figure 1.

Positive self-perception was significantly increased from baseline to Phase 3 of the intervention program. Moreover, a significant effect on mental health problems of children with cancer, as reported by participants themselves (*F* (1, 13) = 19.5, *p* = 0.001), was also observed. From baseline to Phase 3 of the intervention program, there was a significant decrease in mental health problems reported by participants. The art therapy intervention also had a statistically significant effect on the negative self-perception of children with cancer (*F* (1, 13) = 99.11, *p* = 0.000). From baseline to Phase 3 of the intervention program, there was a significant decrease in negative self-perception. The intervention had a statistically significant effect on the overall self-perception of children with cancer (*F* (1, 13) = 605.49, *p* = 0.000). From baseline to Phase 3 of the intervention program, there was a significant improvement in overall self-perception. Finally, the intervention had a statistically significant effect on the mental health problems of children with cancer, as reported by the participants’ attendants: *F* (1, 13) = 21.19, *p* = 0.000. From baseline to Phase 3 of the intervention program, there was a significant decrease in mental health problems. Overall, there was a decrease in mental health problems of children with cancer, demonstrating the efficacy of art therapy.

Overall, the above results showed that the integrative model of art therapy significantly improved the mental health and self-perception of children with cancer. Playdough art therapy also proved to be the most effective of all three sessions in restoring mental health and self-perception.

## 4. Discussion

The current study was the first clinical examination of the impact of an integrative art therapy model on the psychological well-being of pediatric cancer patients. The results showed improvements in positive self-image and a reduction in negative self-perception and mental health problems due to the integrative model.

Firstly, the use of art therapy in combination alleviates mental health problems and improves the quality of life of pediatric cancer patients. This study supports the use of the integrative model in the daily life of cancer patients undergoing painful treatment to reduce mental health problems such as anxiety, depression, hopelessness, low self-esteem, dissatisfaction, lack of motivation, and social problems. Changes due to such integrative approaches were not the focus of previous studies; rather, there was a focus on the effects of art therapies individually. However, these supported the current study by demonstrating a significant decrease in depression and anxiety scores among cancer patients after art therapy, such as calligraphy, drawing, and collage making [41,50,51]. Similarly, physical activity in play therapy improves mental well-being [52]. Group-play therapy is also considered effective in reducing mental health problems such as anxiety in cancer patients [53]. However, the current study explored how the integrative model of art therapy changes the quality of life in pediatric cancer patients by decreasing mental health problems.

Secondly, applying the integrative model significantly increased positive self-image from baseline to phase 3. As discussed earlier, this was the first research to explore the effect of the integrative model. However, many studies support the use of art therapy on cancer patients, such as Thyme et al. [54], who aimed to explore the effect of art therapy on self-image among cancer patients and found a positive change in self-perception post-therapy. According to a systematic review by Bosgraaf et al. [14], fewer than 50% of studies showed an increase in self-esteem and positive self-image of children and adolescents facing cancer as a result of the intervention of art therapy. Another systematic review [55] also supported the effect of art therapies on self-concept or body image in pediatric cancer patients. Similarly, Sanatgar et al. [25] suggested that play therapy is effective in improving social acceptance, hope, and self-image in children facing cancer.

Thirdly, the integrative intervention led to a significant decrease in negative self-perception from baseline to phase 3, as evident from Table 1 and Figure 1. As discussed, this study was the first to explore the combined effect of art therapy and play therapy as an integrative model. However, many studies support the effectiveness of play therapy and art therapy in cancer patients (such as Gazestan et al. [53] and Thomas et al. [56]). Results show a reduction in negative self-image driven by improvements in self-concept and self-esteem. Art therapy also negatively affects body image in cancer patients, improving their quality of life and hope by helping them express their feelings, emotions, and original selves through expressive art [25].

This study would be beneficial in introducing new modes of assessment and management in mental health facilities. Currently, such an unconventional mode of treatment is not in practice, though it holds immense benefits. Also, the arts need no expertise; they rely on the natural way of expression; they are easily implementable in the hospital setting [57,58,59] and are easily accepted by participants and therefore can improve child life services. As a new field in Pakistan, this research will contribute to the limited literature on art therapy in the Pakistani context.

### 4.1. Study Limitations

The current study applied the integrative model to a small sample size, increasing the likelihood that faulty assumptions would be accepted as true. A small sample may also yield unconvincing or potentially deceptive results [60]. A larger, more suitable sample size may have increased the effect size, provided more precise estimates of treatment effects, and provided results generalizable to a broader population. Due to the high dropout rate and difficulty contacting the sample for two months, the sample size was small. Difficulty in recruiting participants due to the sensitive nature of the population and organ amputation led to the exclusion of a large sample; attrition bias is a threat to external validity. In the present research, participants with all forms of cancer were included, which may have affected the study’s validity. A clinically heterogeneous sample makes it harder to identify patterns and effect sizes, increases variability in responses across art modalities, and ultimately hinders a specific conclusion. Even though the staff was informed, many interruptions occurred during the sessions. It was also hard to convince attendants that participation in these sessions would be beneficial for these patients, as people need to consider such modes of treatment as therapeutic. Future research is needed to determine the duration and effectiveness of these therapies, estimate the optimal number of sessions for art therapy, and identify the patients for whom these therapies are more effective. Also, conducting the same research qualitatively, drawing on subjective experiences, would yield more valuable findings.

### 4.2. Implications

The current study’s findings suggest that an integrative model of art therapy and play therapy is important in improving emotional well-being and quality of life in pediatric cancer patients by improving positive self-image and reducing mental health problems. It also suggests that integrative therapies should be incorporated into standard care for pediatric cancer patients. The current study findings carry wider implications for researchers. To build on the present study’s results, future studies may test the integrative art modalities in larger, randomized controlled trials. A randomized controlled trial may be designed to compare outcomes of a group receiving integrative therapies along with standard care, and a control group receiving standard care alone. Moreover, designing these trials over a longer period would compare findings between groups over time and reveal whether the effects of integrative art modalities persist in long-term outcomes, thereby defining specialized patterns of efficacy tailored individually for each participant. In short, the current study explored the effectiveness of an integrative model of art therapy on psychological well-being and quality of life in pediatric cancer patients. Regarding this study, the researchers recommend an integrated model of art therapy and play therapy in general, especially for pediatric cancer patients in active treatment. The findings call for more clinical trials to address this problem.

## 5. Conclusions

To conclude, the current study used an integrated model of art therapy as a promising, feasible, and potentially effective approach not only to enhance self-perception but also to address mental health concerns. Study participants were able to process and heal through their creative expressions, which highlights the therapeutic potential of this approach. Healthcare professionals from pediatric oncology units may consider this creative modality a valuable aid, complementing existing interventions, recognizing its efficacy to foster personal growth and transformation. Further research is necessary to examine the effectiveness of this growth-oriented approach in diverse populations and settings.

## Figures and Tables

**Table 1:** Comparing Study Variables Across Four Phases (N = 14).

Study Variables/Demographics	Baseline	Phase 1	Phase 2	Phase 3	*F* (1, 13)	*η* ^2^
	*M* (*SD*)	*M* (*SD*)	*M* (*SD*)	*M* (*SD*)		
**Patient Reports**						
Mental Health Problems	14.90 (11.11)	11.21 (8.88)	7.21 (6.30)	5.57 (5.04)	19.5 **	0.61
Positive Self-perception	7.79 (3.23)	8.85 (3.25)	9.21 (3.66)	12.28 (3.60)	16.77 **	0.63
Negative Self-Perception	13.86 (3.46)	9.64 (2.27)	9.21 (3.66)	4.50 (3.20)	99.11 ***	0.62
Overall Self-Perception	−6.07 (5.42)	−0.78 (4.13)	18.42 (7.32)	16.78 (0.57)	605.49 ***	0.59
**Attendant Reports**						
Mental Health Problems	24.5 (17.0)	19.86 (14.79)	14.93 (11.57)	7.57 (6.44)	21.19 ***	0.63

** *p* < 0.01, *** *p* < 0.0001.

## Data Availability

The raw data supporting the conclusions of this article will be made available by the authors upon request. The data are not publicly available due to privacy and ethical restrictions.

## Public Involvement Statement

No public involvement in any aspect of this research.

## Guidelines and Standards Statement

This manuscript was drafted against the Transparent Reporting of Evaluations with Nonrandomized Designs (TREND) guidelines [45].

## Abbreviations

The following abbreviations are used in this manuscript:

AT	Art Therapy
ESAS-r	Edmonton Symptom Assessment Scale-Revised
HTP	House Tree Person
